# Bio‐absorbable sealants for reinforcing the pancreatic stump after distal pancreatectomy are critical

**DOI:** 10.1002/jhbp.604

**Published:** 2019-02-10

**Authors:** Christian M. Kühlbrey, Steivan Kasper, Sophia Chikhladze, Gabriel Seifert, Ulrich T. Hopt, Stefan Fichtner‐Feigl, Uwe A. Wittel

**Affiliations:** ^1^ Department of Surgery Faculty of Medicine Clinic for Generalund Visceral Surgery University of Freiburg Medical Center Hugstetter Straße 55 D‐79106 Freiburg Germany

**Keywords:** Adhesives, Animal model, Pancreas, Pancreatic fistula, Polyethylene glycols

## Abstract

**Background:**

Bio‐absorbable sealants are widely used to reduce the rate and severity of postoperative pancreatic fistulas after distal pancreatectomy. However, numerous clinical trials have failed to demonstrate their clinical benefit. We therefore investigated stability and bio‐compatibility of absorbable sealants *in vitro* and *in vivo*.

**Methods:**

*In vitro*, polymerized compounds were incubated in pancreatic juice before their stability was tested. *In vivo*, two compounds were used to seal the pancreatic stump after distal pancreatectomy in nine pigs. Burst pressure of the pancreatic stump, surgical outcome, histology of the pancreatic stump, systemic inflammation, and drain fluid was examined.

**Results:**

Products based on fibrin or collagen were unstable in the presence of active pancreatic enzymes and completely dissolved within 2 h. Sealants using chemical cross‐linking of proteins showed improved stability for 7 days. *In vivo*, application of polyethylenglycol‐based sealant leads to complete closure of the pancreatic duct after 5 days, while a glutaraldehyde‐based sealant prevented physiological closure of the pancreatic main duct.

**Conclusions:**

Many compounds used clinically to reinforce the pancreatic stump after distal pancreatectomy are inadequate due to instability in the presence of pancreatic enzymes. While selected bio‐absorbable sealants inhibited the natural healing of the pancreatic stump, polyethylenglycol‐based sealants should be tested in further clinical trials.

## Introduction

Postoperative pancreatic fistulas (POPF) occur in up to 30% of patients after distal pancreatectomy and largely determine postoperative morbidity 1, 2. Despite substantial efforts, no technique has been developed that significantly reduces the rate of postoperative pancreatic fistulas 3, 4. Surgical techniques appear to have a minor impact on POPF development 4, 5, 6. Reinforcement of the pancreatic stump by an excluded small bowel loop, ligamentum teres plastic, ultrasound dissection of the pancreatic parenchyma, or the use of staplers to dissect the pancreas produce comparable results in terms of POPF formation 7, 8, 9. Desperate to reduce the rate of POPF after distal pancreatectomy, surgeons apply a wide gamut of costly sealants onto the pancreatic stump. Most products are used off‐label and many surgeons do not take into consideration that pancreatic digestive enzymes released from the pancreas are likely to possess proteolytic activity to some degree. Thus, it comes as no surprise that fibrin glue does not reduce POPF in most clinical studies as well as systematic reviews 10, 11, 12. To avoid the weakness of compounds susceptible to proteolysis, non‐absorbable compounds such as acrylamide have been applied as well. These substances are suspected to be prone to bacterial infections in the case of infected fluid collections. Thus, colonized polymers may cause prolonged infections similar to staplerlines.

To avoid sequester formation after reinforcing the pancreatic remnant with sealants, absorbable sealants have been implemented. Especially, the wrapping of the cut surface of the pancreas with a polyglycolic acid mesh significantly reduced rate of clinically relevant POPF after distal pancreatectomy. In an attempt to explain the results from clinical trials examining the reinforcement of the pancreatic stump by sealants and preclinically evaluate commercially available bioabsorbable sealants, we examined the structural stability and *in vivo* biocompatibility of bio‐absorbable sealants applied as liquids for reinforcing the pancreatic stump after distal pancreatectomy.

## Materials and methods

### Human materials

Pancreatic juice was obtained from patients after pancreatoduodenectomy who received a transluminal pancreatic duct drain for other reasons. Informed consent was obtained, and procedures were approved by the local ethics committee (EK Freiburg 106/17). Trypsin activity was determined by colorimetric activity assay (Abcam, Cambridge, UK) before and after activation with enterokinase according to the supplier's instructions. Trypsin activity was determined before co‐incubation with sealants and after 1, 2 and 7 days to confirm persistent enzyme activity. Fresh‐frozen plasma was obtained from the blood bank of the University of Freiburg Medical Center. Only fresh‐frozen plasma that would have been discarded otherwise was used.

### *In vitro* applicability and stability of sealants

The applicability and stability of commercially available bio‐absorbable sealants were tested *in vitro*. A 5‐mm rubber tube was filled with bio‐absorbable sealants, and polymerization was allowed to take place for a minimum of 30 min. Thereafter, the tube was cut into 5 mm long pieces and incubated in pancreatic juice, fresh‐frozen plasma, or saline in 24‐well plates. The following products were tested: Tachoseal (Solid; Baxter Healthcare Corporation, Deerfield, IL, USA), Hemopatch (Solid; Baxter AG, Vienna, Austria), Bioglue (Liquid; CryoLife, Kennesaw, GA, USA), Coseal (Liquid; Baxter Healthcare Corporation, Hayward, CA, USA) and TissuCol (Liquid; Baxter Germany, Unterschleißheim, Germany). The samples were incubated at 37°C for up to 7 days in the presence of 0.1% sodium azide. The stability of the sealant applied in the rubber tubes was tested by burst pressure measurement on day 0, day 1, day 3 and day 7. Burst pressure experiments were performed by inserting the tube fragments into another tube system which was connected to a 50‐ml syringe. The pressure in the tube system was continuously monitored by a handheld digital pressure gauge (Keller, Zürich, Switzerland). The maximum pressure before loss of resistance was automatically recorded (Fig. S1). A minimum of six probes per sealant and time point were examined. In cases of probes that did not withstand any pressure, burst pressure was noted as 0 mmHg. Means and standard error of the mean were calculated. Based on the *in vitro* findings, products for *in vivo* application were chosen.

### *In vivo* stability and biocompatibility

Nine female 4‐month‐old German Landrace pigs (23–29 kg) underwent distal pancreatectomy after approval of the local animal healthcare committee (Thüringer Landesamt für Verbraucherschutz; Reg.‐Nr. 08‐004/14). Pigs were randomized into three groups: in three animals, the pancreatic stump was left untreated without any attempt of closure or sealing; in three animals, Bioglue was applied onto the pancreatic stump; and in three animals, the pancreatic remnant was sealed with Coseal.

After overnight fasting with free access to water, general anesthesia was induced with Azaperone (2 mg/kg i.m.; Jannsen, Beerse, Belgium) and ketamine 10% (20 mg/kg i.m.; Serumwerk Bernburg AG, Bernburg, Germany) for initial intramuscular sedation. Atropine (0.02–0.1 mg/kg body weight; B. Braun Melsungen AG, Melsungen, Germany) was injected subcutaneously. Subsequently, venous access was obtained, and the first blood sample was taken. Endotracheal intubation preceded mechanical ventilation. Anesthesia was maintained with isoflurane (1–2 vol.%) and fentanyl (0.02–0.03 mg/kg/h; Jannsen), midazolam (0.15–0.35 mg/kg to 1 mg/kg; Hexal AG, Holzkirchen, Germany) intravenously. A single‐shot perioperative antibiotic prophylaxis (Enrofloxacin 5%, 0.1 ml/kg; Bayer AG, Leverkusen, Germany) was administered prior to surgical procedures. Heart rate and blood oxygen were monitored continuously. The abdomen was scrubbed with betadine solution, and subsequently, sterile drapes were applied in a standard fashion. Four trocars were placed, and the pancreatic tail was mobilized from adjacent tissue laparoscopically. A mini‐laparotomy was performed and the pancreatic tail was resected approximately 2 cm left of the venous confluens by scalpel (Fig. S2). Hemostasis was obtained directly with hemostasis sutures (5‐0 PDS; Ethicon, Somerville, NJ, USA) if necessary after 1 min of gentle manual compression of the pancreatic stump. Subsequently, the pancreatic stump was left open or closed using a bio‐absorbable sealant according to randomization. A silicon drain was placed close to the pancreatic stump (Blake Silicone Drain; Ethicon) and connected to a bag that was placed in a pocket of a tight‐fitting jacket. Postoperative analgesia was performed with intramuscular injection of meloxicam (0.4 mg/kg body weight, Metacam; Boehringer Ingelheim, Ingelheim am Rhein, Germany).

Animals had free access to food and water as of the evening of the operation.

Daily blood samples were taken from the jugular vein. Drain output and drain amylase concentration were determined daily as well. After 5 days, animals were killed under general anesthesia. The pancreatic head was excised completely, duodenotomy was performed, and a 22G venous catheter was inserted in the pancreatic papilla. Burst pressure of the pancreatic duct system was performed similar to *in vitro* burst pressure measurements. Thereafter, the pancreatic stump was dissected and formalin fixed or frozen and stored at −80°C until further processing.

### Determination of inflammatory mediators and leukocyte function

Inflammation‐related mediators were determined in serum samples by ELISA. Porcine DuoSet ELISA kits for C‐reactive protein (CRP) were obtained from R&D Systems (Minneapolis, MN, USA). Elisa DuoSets were performed on 96‐well plates and used according to the supplier's instructions. Each sample was quantified in duplicate. Average values and standard error of the mean were calculated.

To determine perioperative leukocyte function, whole blood was incubated with white blood cell stimulants. Whole blood with added heparin was diluted 1:10 with RPMI medium 1640 (Gibco, Life Technologies, Carlsbad, CA, USA) supplemented with penicillin/streptomycin (Biochrom, Berlin, Germany). Diluted blood samples were stimulated with 5 ng/ml Concanavalin A (ConA; Sigma, Steinheim, Germany), 0.5 ng/ml Staphylococcal Enterotoxin B (SEB; Sigma) or 10 ng/ml lipopolysaccharide (LPS; Sigma) and incubated for 24 h at 37°C. After 24 h, supernatants were frozen at −80°C until further processing by ELISA. ELISAs were performed for IL‐6 and IFN‐gamma using porcine DuoSet ELISA kits (R&D Systems) according to the supplier's instructions in 96‐well plates.

### Immunohistochemistry

For histology and immunohistochemistry, 3 μm sections were cut perpendicular to the pancreatic resection surface and stained with hematoxylin and eosin. Immunohistochemistry was performed for α‐SMA, FGF‐1, Ki67, SMAD2, SMAD4 and NF‐κB p65. For α‐SMA and NF‐κB, slides were boiled in 0.01 M Tris/EDTA pH9 buffer and incubated at 4°C overnight with monoclonal mouse anti‐α smooth muscle actin antibody (ab7817; Abcam) in a dilution of 1:100 or NF‐κB in a dilution of 1:500 diluted in antibody diluent with background reducing components (Dako, Glostrup, Denmark) for 18 h at 4°C. Slides were then incubated with the EnVision System‐HRP‐labeled polymer anti‐mouse (Dako). The color reaction was obtained by DAB plus Substrate kit (Invitrogen, Carlsbad, CA, USA) before counterstaining with hematoxylin. FGF‐1, Ki67, SMAD2 and SMAD4 staining were performed after boiling the slides in 0.01 M sodium citrate buffer (pH6) with 0.05% Tween 20 prior to incubation with the following antibodies: polyclonal rabbit anti fibroblast growth factor 1 (AA 12‐151; Antibodies‐online, Aachen, Germany) in a 1:25 dilution, polyclonal rabbit anti‐Ki67 (ab15580; Abcam) in a 1:500 dilution, polyclonal rabbit anti‐Smad2 (ab47083; Abcam) in a 1:50 dilution, and recombinant monoclonal rabbit anti‐Smad4 (ab40759; Abcam) in a 1:100 dilution. All antibodies were diluted in antibody diluent (Dako). After washing with TBST, slides were treated with EnVision System‐HRP‐labeled polymer anti‐rabbit (Dako). Antibody binding was detected using the DAB plus substrate kit (Invitrogen) and then lightly counterstained with hematoxylin.

### Cell culture and MTT assay

Human foreskin fibroblast cells were cultivated in DMEM with 10% FBS (Life Technologies). 3 × 10^4^ HFF cells were seeded homogenously in 24‐well cell culture plates. After 24 h, Bioglue or Coseal was polymerized in cell culture inserts and the inserts were inserted into the cell culture. At that point and daily thereafter, MTT assays were performed by adding 50 μl of a 5 mg/ml MTT (3‐4,5‐dimethylthiazol‐2‐yl)‐2,5‐diphenyl tetrazolium bromide; Sigma) solution to the cell culture which then was incubated for 4 h at 37°C. Thereafter, the medium was removed and 250 μl isopropanol/0.04N hydrochloric acid was added to each well. Plates were put on a shaker for 20 min, and absorbance was monitored at 570 nm and 650 nm. Relative absorbance to day 0 was calculated.

### Expression of Apoptosis related genes

2 × 10⁵ HFF cells per well were seeded into 6‐well plates in 2.5 ml DMEM medium. After 24 h, filter inserts with already polymerized Bioglue, Coseal or empty cell culture inserts were placed in the wells. After 12 h, cells were washed two times with PBS and cell lysis was initiated with 350 μl RLT Plus buffer (Qiagen, Hilden, Germany). The lysate was directly transferred into a QIAshredder spin column. RNA isolation was performed with the RNeasy Plus Mini Kit (Qiagen) according to the supplier's instructions. 1 μg RNA was transcribed into cDNA, and an RT^2^ Profiler PCR Array Pig Apoptosis was performed on a Roche light cycler 480 in a 384‐well format. Data analysis was performed using RT^2^ Profiler PCR Array Data Analysis version 3.5 online tool (Qiagen SABiosciences, Hilden, Germany). Data were expressed as fold regulated compared to control wells without bio‐absorbable sealant.

### Statistical analysis

Data on animal experiments are presented in percent of initial value due to strong baseline value differences in individual animals at the beginning of the experiment. Data are presented as mean and standard error of the mean. Continuous variables were analyzed using ANOVA followed by Dunnett's post hoc test. The Mann–Whitney *U*‐test was applied where appropriate. Statistical significance was defined as a *P*‐value of less than 0.05.

## Results

### *In vitro* stability of sealants

Bio‐absorbable materials were incubated in activated pancreatic juice for up to 7 days at 37°C. *In vitro*, both hemostatic patches completely dissolved in pancreatic juice after 2 h while in saline their structure was unaltered (Fig. S3). Due to the rapid dissociation of the patches tested further experiments with collagen or fibrin‐based products were not possible.

**Figure 1:**
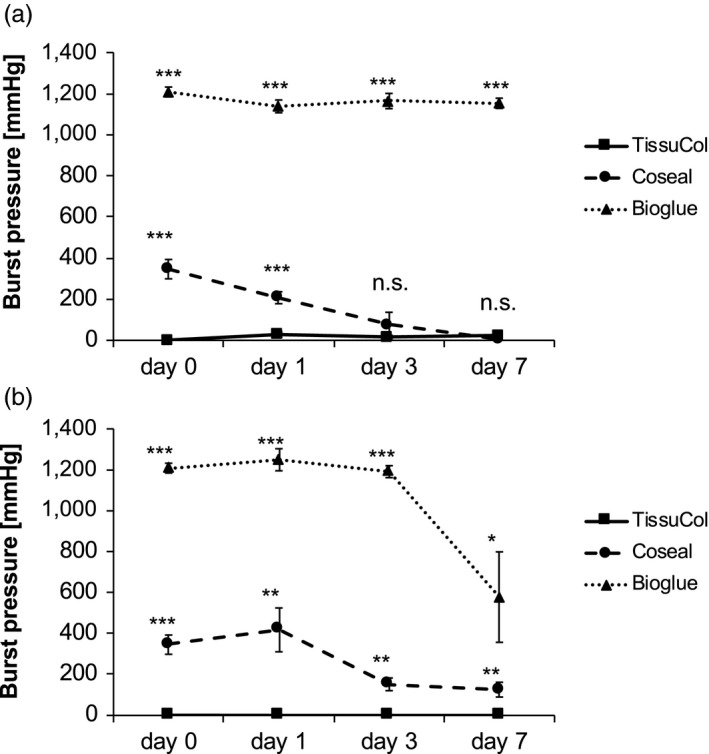
(**a**) Burst pressure after incubation of polymerized liquid bio‐absorbable adhesive in human plasma. (**b**) Burst pressure after incubation of polymerized liquid bio‐absorbable adhesive in pancreatic juice. Bioglue and Coseal both lost part of their stability after 7 days in pancreatic juice. In contrast to TissueCol, which quickly lost its integrity, the other two compounds showed sufficient stability to reasonably reinforce the pancreatic stump for up to 7 days after distal pancreatectomy. (***P < 0.001, **P < 0.01, *P < 0.05)

The three liquid adhesives were tested by mixing two components prior to insertion into rubber tubes and incubation in pancreatic juice, saline or plasma. Fibrin glue was difficult to apply, and incomplete polymerization due to incomplete mixing initially leads to low burst pressures. After incubation in human plasma, stability increased, but burst pressure was still significantly lower (Fibrin Glue 28 ± 11 mmHg, Coseal 207 ± 29 mmHg, Bioglue 1138 ± 31 mmHg; *P* < 0.001). While fibrin dissociated completely in pancreatic juice before day 1, Bioglue and Coseal maintained stability even in activated pancreatic juice until day 7 (Fig. 1). With both products, burst pressure remained almost unaltered during the first 3 days with a subsequent decrease thereafter. After 1 week, burst pressure values were reduced to slightly less than half of initial values (Bioglue 578 ± 221 mmHg; Coseal 126 ± 36 mmHg). Further experiments were conducted with the two compounds showing sufficient *in vitro* stability.

### *In vivo* application of bio‐absorbable sealants

All animals underwent distal pancreatectomy without intraoperative complications, and all animals survived 5‐day follow‐up as intended. The median operative time was 78 min (50–110 min). One animal developed a trocar hernia with consecutive bowel obstruction. The hernia was detected on day 5 and did not interfere with treatment protocol. One animal removed its abdominal drains on postoperative day (POD) 1 requiring surgical replacement in general anesthesia.

**Figure 2:**
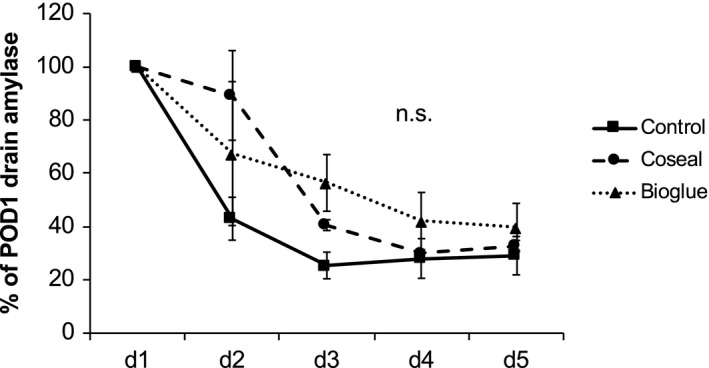
Change in drain amylase concentration. Drain amylase concentration after distal pancreatectomy decreased between POD 1 and POD 3. There was no difference among the groups. Interestingly, the drain amylase concentration in the control group, in which the pancreatic remnant was left unsealed, was not significantly elevated

Measurement of daily drain output and drain amylase concentrations was not influenced by the treatment but declined throughout the experiments in all three groups (Fig. 2). Serum amylase concentrations were affected by treatment of the pancreatic remnant (Fig. S4). In animals treated with Bioglue, serum amylase concentration was elevated on day 1 (control 109 ± 3% of preoperative value, Bioglue 178 ± 37%, not significant) indicating pancreatic acinar cell damage. Coseal‐treated animals showed a gradual increase in serum amylase until POD 5 that was significantly elevated throughout the postoperative course compared to control animals.

**Figure 3:**
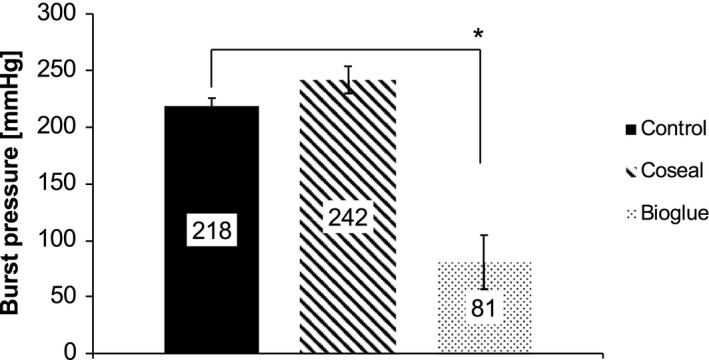
Burst pressure of the pancreatic duct 5 days after distal pancreatectomy revealed spontaneous occlusion in untreated animals. Bioglue‐treated animals showed significantly lower burst pressure compared to control and Coseal‐treated animals which was induced by an inhibition of the natural occlusion of the pancreatic duct observed in both other groups

Five days after distal pancreatectomy, the remaining pancreas was removed, and the pancreatic duct was cannulated. Leak of the pancreatic duct and stability of duct occlusion were examined by determining burst pressures. Animals with untreated pancreatic remnants showed complete occlusion of the pancreatic duct that withstood pressure of up to 218 ± 7 mmHg (Fig. 3). The pancreatic duct system of Coseal‐treated animals also withstood high pressures of 242 ± 12 mmHg. In contrast to these findings, the treatment of the pancreatic stump with Bioglue significantly reduced maximum burst pressure to 81 ± 24 mmHg (*P* < 0.05). Bioglue showed a loose adhesion to the pancreatic parenchyma, and in contrast to the other animals, the main pancreatic duct could clearly be identified in the resection plane on POD 5 (Fig. S5). The application of Bioglue was disrupting spontaneous duct occlusion.

**Figure 4:**
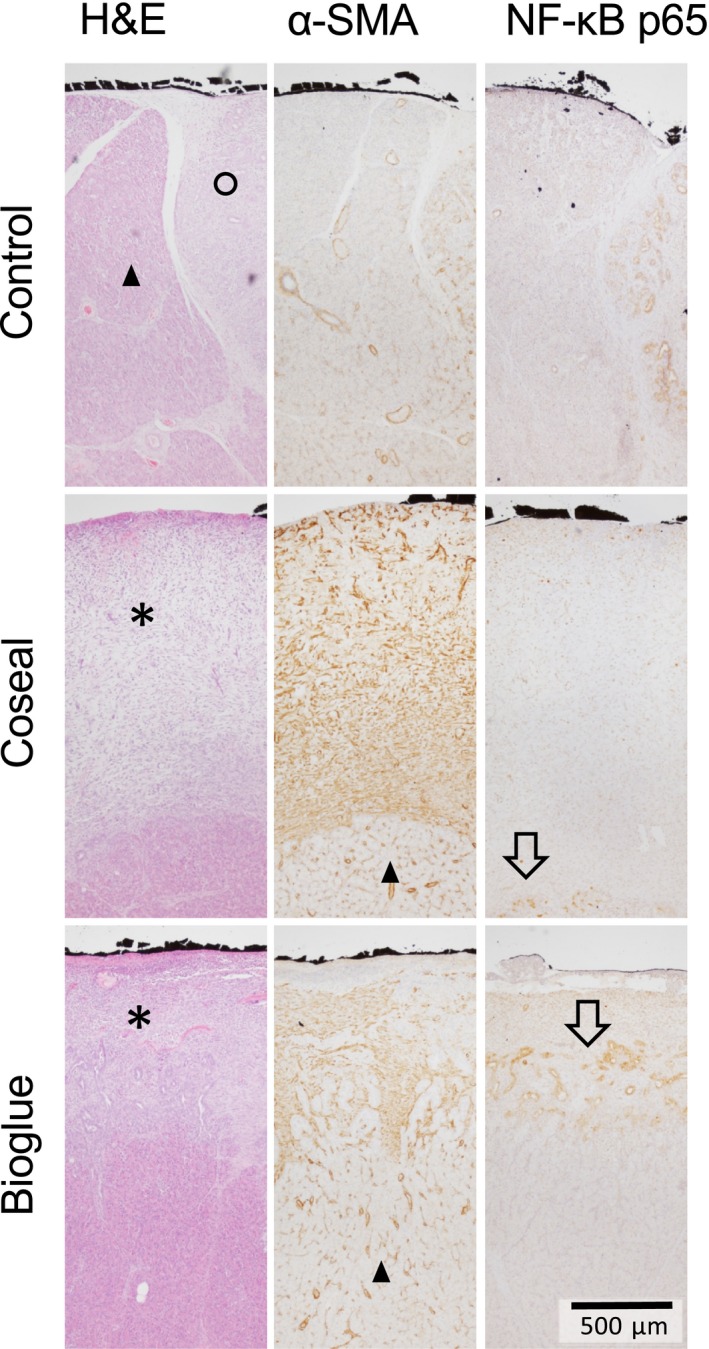
Histological presentation of the pancreatic dissection plane (black ink – surface of pancreatic remnant). H&E: the alterations of pancreatic parenchyma in untreated animals were fewer than in sealant treated animals. Here, we observed a cell‐rich layer in‐between acinar cells and the sealant (asterisk). α‐SMA: α‐SMA staining indicated increased activation of pancreatic stellate cells in the intermediate layer that was thinner in Bioglue‐treated animals. NF‐κB: the inflammatory response was most pronounced in animals with a Bioglue‐treated pancreatic stump. NF‐κB was expressed in ductal structures possibly identifying areas of acinar‐to‐ductal metaplasia

Histopathological evaluation of the dissection plane showed no differences in Ki‐67, Vimentin, SMAD2, SMAD4 and FGF expression (data not shown). Both sealants induced a cell‐rich layer between the sealant and pancreatic acinar cells, while control animals had only minor histological alterations in this area. In control animals, acinar cell reduction and acinar‐to‐ductal metaplasia were observed (Fig. 4, circle). Generally, the cell‐rich layer in Bioglue‐treated animals appeared to be thinner than in Coseal‐treated animals (Fig. 4, asterisk). Stellate cell activation was detected by α‐SMA staining. α‐SMA expression was pronounced in the above mentioned cell‐rich layer between acinar cells (Fig. 4, triangle) and sealants. We observed increased expression of phosphorylated NF‐κB, indicating an inflammatory response in duct‐like structures between acinar cells and the fibrotic layer. This may point to acinar‐to‐ductal metaplasia (Fig. 4, arrows). In Coseal‐treated animals and controls, these areas appeared limited to few, duct‐like structures.

**Figure 5:**
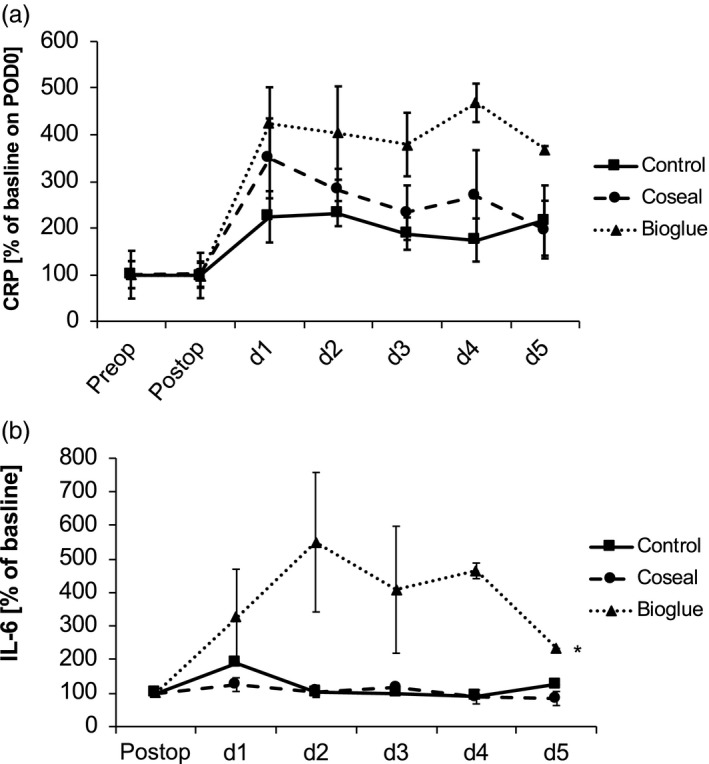
(**a**) Change in C‐reactive protein (CRP) concentrations. CRP was elevated after distal pancreatectomy in pigs. The elevation was noted throughout the entire experiment. The method of reinforcing the pancreatic stump had in impact on the CRP concentration. Animals treated with Bioglue showed increased CRP concentration until POD 5. (**b**) IL‐6 concentrations after stimulation with lipopolysaccharide (LPS). Cell sensitivity was even more pronounced when leukocytes were activated with LPS and IL‐6 release was quantified. In these experiments, Bioglue‐treated animals showed increased IL‐6 release upon LPS stimulation (*P < 0.05)

Animals treated with Bioglue developed significantly higher CRP values throughout the entire postoperative course. This appeared to be specific to Bioglue since serum concentrations of CRP in Coseal‐treated animals was not increased (Fig. 5a). Stronger systemic response to the application of Bioglue versus Coseal onto the pancreatic stump was further supported by differences in sensitivity of peripheral leukocytes to activating stimuli. When leukocytes were stimulated with ConA and LPS increased sensitivity of white blood cells was observed in Bioglue‐treated animals. In these experiments, *ex vivo* stimulation of lymphocytes with ConA induced significant increase in interferon gamma in the cell culture supernatant (Fig. S6) and the stimulation of phagocytes with LPS induced increased IL‐6 release from phagocytes of Bioglue‐treated animals (Fig. 5b). Treatment with the polyethylene glycol‐based sealant did not show systemic inflammatory effects.

**Figure 6:**
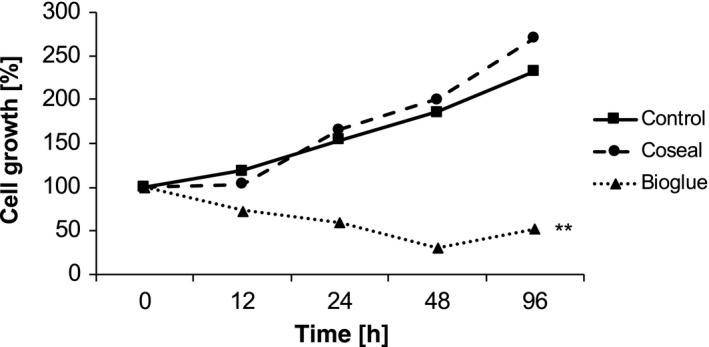
*In vitro* fibroblast cell growth. Fibroblast growth of human foreskin fibroblasts incubated with Coseal and Bioglue without direct contact to the sealant. Soluble components of polymerized Bioglue not only induced significant reduction in cell growth but also reduced the number of viable fibroblasts *in vitro*, explaining the impaired healing of the pancreatic stump after Bioglue application (**P < 0.01)

**Figure 7:**
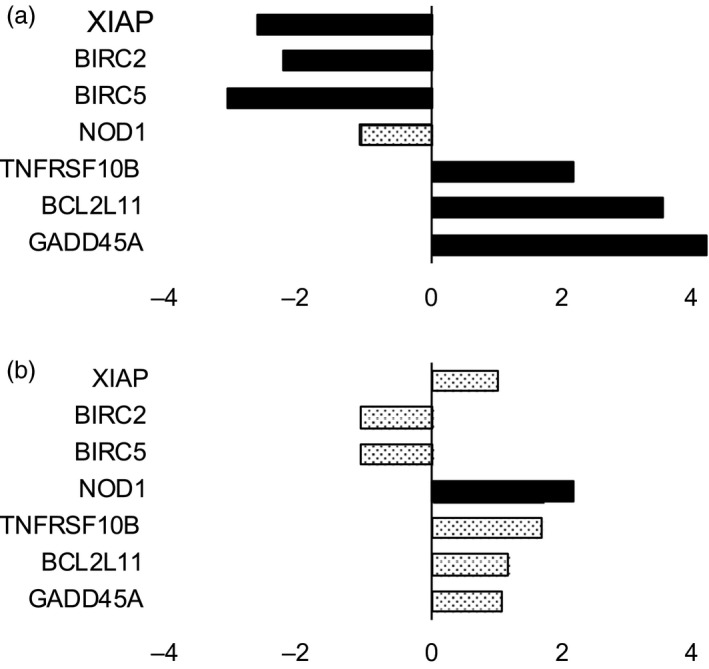
(**a**) Relative expression of apoptosis associated genes – Bioglue. (**b**) Relative expression of apoptosis associated genes – Coseal. The reduction of fibroblasts in the presence of soluble factors of bioglue is most likely due to apoptosis. Expression of anti‐apoptotic genes XIAP, BIRC2, BIRC5 was reduced pro‐apoptotic genes TNRFST10B, BCL2L11, and GADD45A were expressed at higher levels. In Coseal‐treated fibroblasts, only NOD1 showed a more than 2‐fold change in expression, possibly due to its role as an intracellular pattern recognition receptor

To examine the effect of solved components of the sealants on human fibroblasts, we examined the effect of Bioglue and Coseal conditioned medium on the growth and gene expression of human fibroblasts. While the presence of polymerized Coseal had no effect on cell viability and cell growth, the presence of polymerized Bioglue decreased cell viability significantly (Fig. 6). Gene expression profiling revealed that Bioglue induced apoptosis since Bioglue upregulated genes in human fibroblasts associated with apoptosis, such as TNFSF10B and downregulated, e.g. BIRC2 and BIRC5 (Fig. 7). In contrast, Coseal did not induce significant changes in apoptosis‐related genes except NOD1, which is involved in other functions as well.

## Discussion

Postoperative pancreatic fistulas after distal pancreatectomy develop frequently. Bioabsorbable sealants capable of reducing the rate and duration of postoperative pancreatic fistulas would improve the postoperative course of these patients. However, many attempts to seal the pancreas have failed in clinical trials. Our data demonstrate that substances used to reinforce the pancreatic remnant after distal pancreatectomy must be chosen with care. Since chemically non‐cross‐linked substances are susceptible to proteolytic enzyme cleavage released from pancreatic parenchyma reasonable mechanical reinforcement cannot be expected from these substances. In our experiments, they dissolved within hours in pancreatic juice. This explained results from clinical meta‐analyses that show no clinical benefit for the application of fibrin‐based sealants 11, 12, 13, 14, 15.

In our study, two substances with acceptable *in vitro* stability were examined. Bioglue is a bio‐absorbable sealant mainly used in cardiovascular and thoracic surgery for sealing graft anastomoses and bronchial closures. It consists of glutaraldehyde and bovine serum albumin molecules that covalently cross‐link with each other and with tissue proteins. Absorption of the sealant is believed to be very slow due to the high rate of cross‐linking. The second substance, Coseal, is a sealant that is developed and used in cardiovascular and thoracic surgery. This sealant contains two polyethylene glycol polymers that cross‐link rapidly with each other and proteins of the target tissue 16.

Due to their chemical cross‐linking, the stability of both substances even when in contact with active pancreatic proteolytic enzymes was sufficient up to 7 days *in vitro* in burst pressure experiments. In fact, burst pressure was only reduced by half of the initial values after 7 days. However, chemical reactions occurring during polymerization and the possibility of substances diffusing from polymerized products require *in vivo* investigation of both compounds.

A significant reduction of burst pressure compared to untreated animals was observed in Bioglue‐treated animals due to a lack of occlusion of the pancreatic duct and a loss of adhesion of Bioglue from the pancreatic stump. This indicated local toxicity of Bioglue that inhibited natural occlusion of the pancreatic stump. Also, an increase in systemic inflammation in Bioglue‐treated animals was observed. These systemic effects of Bioglue may be explained by compounds released from the polymerized product and direct interaction with immune cells or by increased infectious complications that have been observed with Bioglue application 17, 18. Local effects of Bioglue were induced by an interaction of the sealant with pancreatic tissue initiating increased local inflammation as NF‐κB staining is increased comparable to acute pancreatitis where acinar cell damage induced nuclear NF‐κB upregulation 19. Adverse effects of Bioglue have also been observed in other types of tissue. When Bioglue was applied for sealing liver or lung tissue, Bioglue even induced cell necrosis 17, 20, 21. These effects were believed to be due to substantial amounts of glutaraldehyde released from polymerized Bioglue 17. *In vivo*, the effects of glutaraldehyde may be quenched by the surplus of binding tissue proteins, but our observations did not corroborate this hypothesis. The increase in systemic amylase was initiated by local pancreatic acinar cell damage induced by application of Bioglue. It was likely due to the same mechanisms that are responsible for Bioglue's interference with peripheral nerves 22. Increased sensitivity of pancreatic tissue to sealants is not limited to glutaraldehyde. Cyanoacrylate derivates showed similar adverse effects, most likely due to local toxicity 22 but the alterations induced by Bioglue were not observed in animals with Coseal reinforced pancreatic stump. Neither systemic effects nor the decrease in burst pressure after *in vivo* Coseal application was observed. In fact, burst pressures in animals treated with Coseal were identical to animals with spontaneous duct occlusion after distal pancreatectomy. The reason for this observation lies in the different chemistry of polymerization where polyethylenglycols do not appear to display similar toxic effects on the pancreatic tissue as glutaraldehyde.

Our experiments do not support the clinical application of additional sealing of the pancreatic stump after distal pancreatectomy. While Bioglue increases local and systemic inflammatory reactions leading to impaired healing of the pancreatic stump, this was not observed with Coseal. Due to the spontaneous closure of pancreatic fistulas in porcine and rodent animal models, it is not possible to generate experimental evidence in terms of POPF formation. Clinical trials with substances not inducing detectable adverse events in animal experiments will be the only possibility to examine their effect on POPF development and positive clinical data on scaffold‐based polyethylene glycol products support this strategy 23. In future, clinical trials examining the effect of further absorbable sealants reinforcing stapler lines after distal pancreatectomy on POPF formation with substances with reasonable biocompatibility will have to be performed.

## Conflict of interest

None declared.

## Author contributions

C.K., U.W. and U.H. designed the study, C.K., U.W. and S.K. acquired the data. C.K. and S.K. analyzed the data, C.K., U.W., S.C. and S.K. interpreted the data and performed statistical analysis. C.K. and U.W. wrote the manuscript. G.S., S.F., S.C. and U.H. helped revise the manuscript. All authors discussed the data and the analysis methods and contributed to the manuscript.

## Supporting information


**Figure S1**. Burst pressure experiments were performed by inserting the tube fragments into another tube system which was connected to a 50 ml syringe.

**Figure S2**. Pancreas anatomy in the porcine.

**Figure S3**. Incubation of matrix bound hemostasis patches in pancreatic juice or saline.

**Figure S4**. Serum amylase concentration in Coseal treated animals was significantly higher than in control animals, but not significantly higher than in Bioglue animals.

**Figure S5**. Macroscopic appearance 5 days after distal pancreatectomy when the pancreatic remnant was sealed with Bioglue.

**Figure S6**. Immune cells of animals treated with Bioglue showed sensitivity.
