# Supplementary figures and images for: Bio‐absorbable sealants for reinforcing the pancreatic stump after distal pancreatectomy are critical
Source: J Hepatobiliary Pancreat Sci. 2019 Feb 10;26(3):96–103. doi: 10.1002/jhbp.604 (PMC6593819; doi:10.1002/jhbp.604)

Online Supplemental Fig. 1

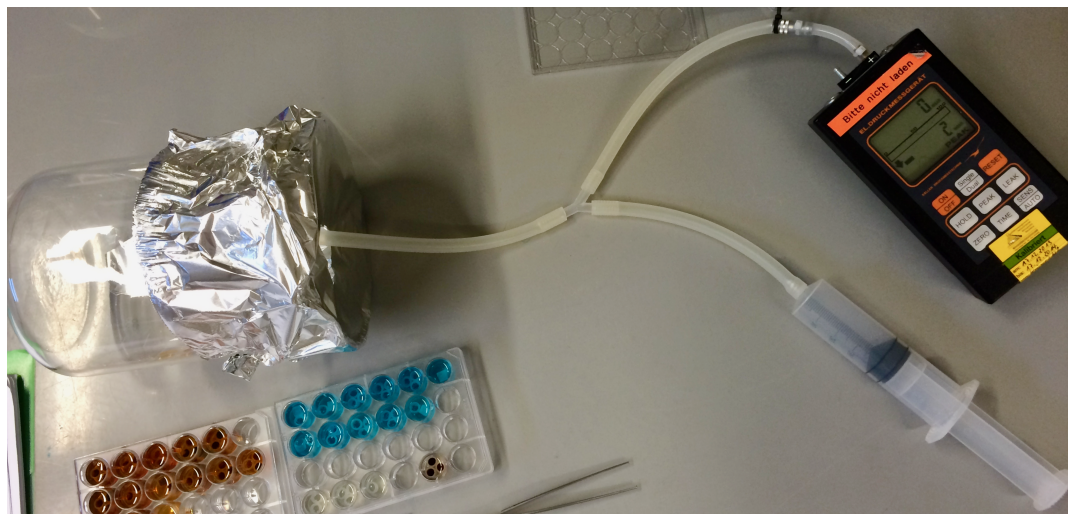

Supplement: Supplementary file 1 — Figure S1. Burst pressure experiments were performed by inserting the tube fragments into another tube system which was connected to a 50 ml syringe. [file JHBP-26-96-s001.pdf]

Online Supplemental Fig. 2

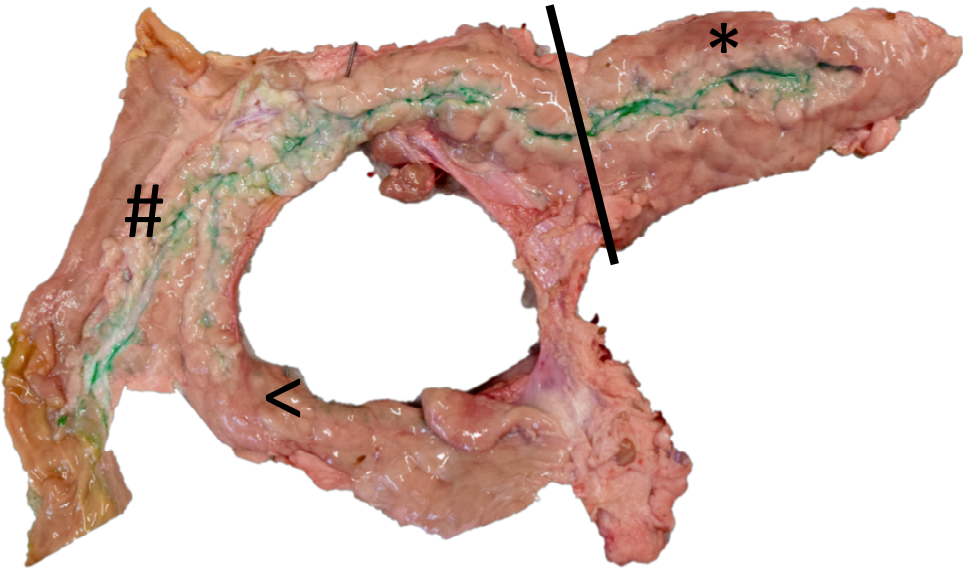

Supplement: Supplementary file 2 — Figure S2. Pancreas anatomy in the porcine. [file JHBP-26-96-s002.pdf]

Online Supplemental Fig. 3

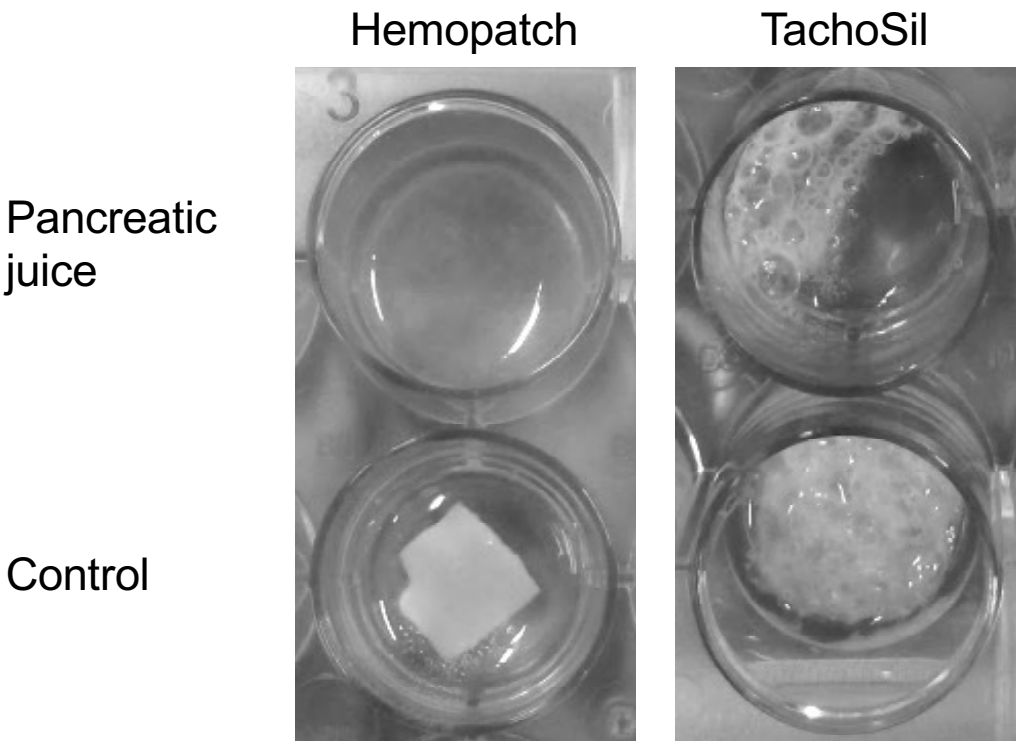

Supplement: Supplementary file 3 — Figure S3. Incubation of matrix bound hemostasis patches in pancreatic juice or saline. [file JHBP-26-96-s003.pdf]

Online Supplemental Fig. 4

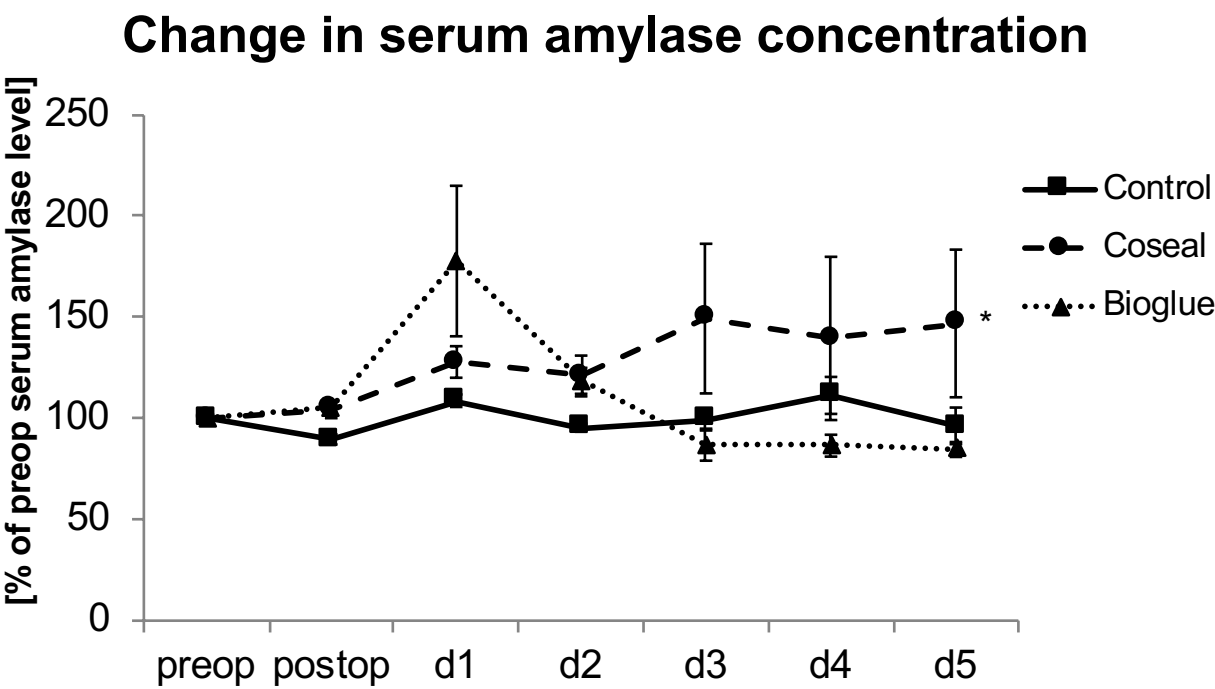

Supplement: Supplementary file 4 — Figure S4. Serum amylase concentration in Coseal treated animals was significantly higher than in control animals, but not significantly higher than in Bioglue animals. [file JHBP-26-96-s004.pdf]

Online Supplemental Fig. 5

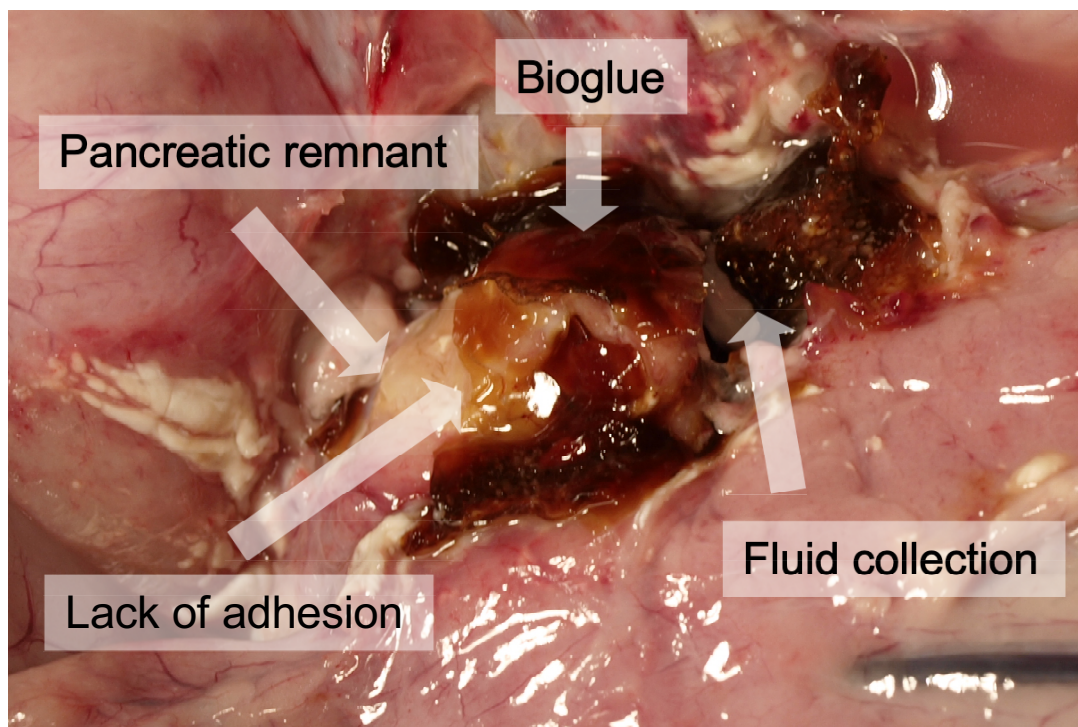

Supplement: Supplementary file 5 — Figure S5. Macroscopic appearance 5 days after distal pancreatectomy when the pancreatic remnant was sealed with Bioglue. [file JHBP-26-96-s005.pdf]

Online Supplemental Fig. 6

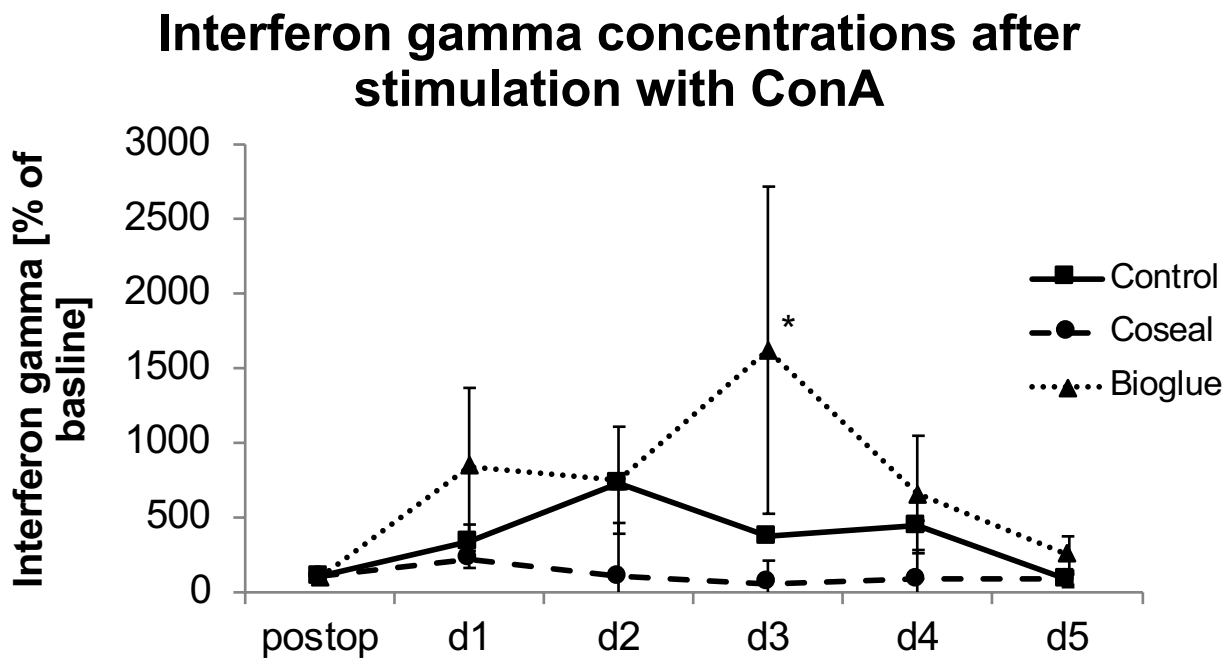

Supplement: Supplementary file 6 — Figure S6. Immune cells of animals treated with Bioglue showed sensitivity. [file JHBP-26-96-s006.pdf]
